# The Use of Cost-Effectiveness Thresholds for Evaluating Health Interventions in Low- and Middle-Income Countries From 2015 to 2020: A Review

**DOI:** 10.1016/j.jval.2021.08.014

**Published:** 2022-03

**Authors:** Joseph Kazibwe, Adrian Gheorghe, David Wilson, Francis Ruiz, Kalipso Chalkidou, Y-Ling Chi

**Affiliations:** 1 Global Health and Development Group, School of Public Health, Imperial College London, Norfolk Place, London, England, UK; 2 International Decision Support Initiative, Center for Global Development, London, England, UK; 3 MRC Centre for Global Infectious Disease Analysis and the Abdul Latif Jameel Institute for Disease and Emergency Analytics, School of Public Health, Imperial College London, England, UK; 4 Bill & Melinda Gates Foundation, London, England, UK

**Keywords:** 1 to 3× gross domestic product per capita, cost-effectiveness thresholds, *decision* rules, low- and middle-income countries

## Abstract

**Objectives:**

Evidence-informed priority setting, in particular cost-effectiveness analysis (CEA), can help target resources better to achieve universal health coverage. Central to the application of CEA is the use of a cost-effectiveness threshold. We add to the literature by looking at what thresholds have been used in published CEA and the proportion of interventions found to be cost-effective, by type of threshold.

**Methods:**

We identified CEA studies in low- and middle-income countries from the Global Health Cost-Effectiveness Analysis Registry that were published between January 1, 2015, and January 6, 2020. We extracted data on the country of focus, type of interventions under consideration, funder, threshold used, and recommendations.

**Results:**

A total of 230 studies with a total 713 interventions were included in this review; 1 to 3× gross domestic product (GDP) per capita was the most common type of threshold used in judging cost-effectiveness (84.3%). Approximately a third of studies (34.2%) using 1 to 3× GDP per capita applied a threshold at 3× GDP per capita. We have found that no study used locally developed thresholds. We found that 79.3% of interventions received a recommendation as “cost-effective” and that 85.9% of studies had at least 1 intervention that was considered cost-effective. The use of 1 to 3× GDP per capita led to a higher proportion of study interventions being judged as cost-effective compared with other types of thresholds.

**Conclusions:**

Despite the wide concerns about the use of 1 to 3× GDP per capita, this threshold is still widely used in the literature. Using this threshold leads to more interventions being recommended as “cost-effective.” This study further explore alternatives to the 1 to 3× GDP as a decision rule.

## Highlights

• The assessment of cost-effectiveness of interventions ex ante can ensure that cost-effective interventions that highly benefit population health are consistently prioritized over interventions that offer comparatively less value.
• Cost-effectiveness thresholds (CETs) are essential in determining whether an intervention under consideration is cost-effective or not; nevertheless, there is no international consensus on how they should be determined by countries.
• The World Health Organization has distanced itself from the use of 1 to 3× gross domestic product (GDP) per capita per disability-adjusted life-year averted (earlier recommended by World Health Organization-CHOICE) as a CET but they remain the most popular and commonly used thresholds.
• More than a third of studies apply the 1 to 3× GDP per capita CET use 3× GDP per capita per disability-adjusted life-year averted yet 3× GDP per capita is a lot higher than other CET.
• Using 1 to 3× GDP per capita as a CET has resulted in a higher proportion of study interventions being found as cost-effective in comparison with studies where other types of CET are used. This may lead to a loss of would-be health gains from deployed resources for health.
• There is an urgent need to move away from 1 to 3× GDP per capita as a CET and a more appropriate replacement should be found.

## Introduction

The regular assessment of the cost-effectiveness of interventions ex ante can ensure that cost-effective interventions that highly benefit population health are consistently prioritized over interventions that offer comparatively less value in coverage decisions.^1^ Cost-effectiveness analyses (CEAs) compare the relative costs and health gains (eg, measured through disability-adjusted life-years [DALYs] or quality-adjusted life-years) from the introduction of an intervention. Central to CEA is the incremental cost-effectiveness ratio (ICER), which summarizes the expected incremental costs to the incremental benefits of a given intervention or program compared with an alternative (eg, standard/usual care).^2^ ICERs can be compared with a decision rule called the cost-effectiveness threshold (CET). If the ICER is below the CET, then the intervention or program under consideration is deemed cost-effective, and if it is above the threshold, then the intervention or program is regarded as not cost-effective. A report by the World Health Organization (WHO) indicates that critically assessing interventions and selecting optimal intervention mix in countries could lead to achieving the same health gains with between 16% and 99% of current spending.^3^

There is no global CET or consensus on how countries should develop their CET to inform the utilization of public/pooled resources for health (see Appendix 1 in Supplemental Materials found at https://doi.org/10.1016/j.jval.2021.08.014 for information on methods).^4^ Ideally, CETs should be contextualized to local health constraints, in particular with regard to available resources or population preferences. For instance, what defines a cost-effective service in the United-States (where public spending on health per capita in 2019 was $9386^5^) will be very different from what may be cost-effective in Uganda (where public spending on health per capita in 2017 was $24^6^). Countries with a local CET include Ireland and England,^7^ but with the exception of a few (eg, Thailand^8^), local thresholds are rare in low- and middle-income countries (LMICs). Because of this gap, CETs that have been used internationally such as the 1 to 3× gross domestic product (GDP) per capita rule have grown in importance, both in research and for consideration by public payers.

For the good part of the last decade, GDP-based thresholds have been considered unrealistic because they were based on flawed technical evidence^9^ in addition to being completely disconnected with health opportunity costs or local budgets constraints.^10^^,^^11^ One big concern is that GDP-based thresholds are too easily attainable^12^ and therefore push for the inclusion of interventions that may not be affordable (or cost-effective from perspective of health opportunity costs).^13^ This may lead to misallocation of resources and cause losses in health.^9^ It is worth noting that, despite those increasing concerns (including from staff at the WHO), the 1 to 3× GDP threshold is still in use in WHO-CHOICE. A review conducted by Leech et al^2^ found that 66% of the 381 CEAs published in LMICs between 2000 and 2015 use GDP-based thresholds, with an increasing trend over that period (23% did not report any threshold). Nevertheless, Leech et al^2^ looked at the broad categories of GDP-based thresholds, so it is not clear what the exact GDP rule used in the studies they considered is. Specifying the rule clearly is important, because, by definition, 1 to 3× GDP per capita is a 3-fold range.

The aim of this study is 2-fold. First, we seek to document what CETs have been used in published CEAs in detail (eg, whether it is 0.5, 1, 2, or 3× GDP per capita) and, second, how that correlated with the recommendations made by the authors (cost-effective/not cost-effective). Moreover, the period adopted in this review goes beyond the timeframe covered by Leech et al,^2^ which will allow us to compare our results to trends identified by their study, in particular after the growing critique of the GDP-based CET around the cutoff date (Marseille et al^14^ and Bertram et al^11^).

## Methods

This review followed the Preferred Reporting Items for Systematic Reviews and Meta-Analyses guidelines.^15^ This review relies on the Global Health Cost-Effectiveness Analysis (GH CEA) Registry managed by the Center for the Evaluation of Value and Risk in Health at Tufts Medical Center.^16^ The registry is a repository of all studies in English language majorly using the “cost-per-DALY averted” metric to measure the efficacy of health interventions around the globe.^17^^,^^18^ Therefore, it was considered because of its comprehensiveness^17^ and coverage of CEAs in LMICs. We searched the GH CEA Registry on January 6, 2020, for studies published since January 1, 2015. All the studies (indexed in PubMed) that came up were extracted in an excel file and reviewed for inclusion into the study. The GH CEA Registry is organized around interventions, not studies.

Studies that met the following criteria were included: (1) presents cost-effectiveness findings measured in costs per effect outcome such as DALYs and (2) has been conducted within/for a LMIC as categorized by the World Bank as of January 6, 2020. The description of the threshold used and recommendation was extracted manually. In this study, we define a recommendation as the conclusion drawn about cost-effectiveness of an intervention following the use of a given threshold, typically after the “Results” section.

## Results

A total of 232 cost-effectiveness studies published in respective journals between January 1, 2015, and January 6, 2020, were identified from the GH CEA database. Using the inclusion and exclusion criteria, 2 studies were excluded because they focused on high income countries. A total of 230 studies were included in the review and underwent full-text reading and data extraction.

### Characteristics of Studies

The summary of characteristics of the studies that have been assessed and analyzed in this study is presented in Table 1. Both the studies (N = 230) and their respective interventions (N = 713) are considered as most studies looked at >1 intervention.

**Table 1:** Characteristics of the studies and interventions.

Number of studies (N = 230)	Frequency	Percentage
Studies by number of countries studied		
One country	184	80.0
Multiple countries	46	20.0
Regional distribution of studies		
Sub-Saharan Africa	90	39.1
Multiple regions	41	17.8
South Asia	35	15.2
East Asia and Pacific	31	13.5
Latin America and Caribbean	20	8.7
Middle East and North Africa	9	3.9
Europe and Central Asia	4	1.7
Funding body/institution by study		
Foundation	80	34.8
Government	60	26.1
Not mentioned	46	20.0
None	16	7.0
Academic institution	10	4.4
Professional member organization	5	2.2
Manufacturer	4	1.7
Healthcare organization	3	1.3
Others	6	2.6
Disease area		
HIV	31	13.5
TB and malaria	28	12.2
Maternal child health	25	10.9
NCDs except cancer and mental health	21	9.1
Cancer	6	2.6
Mental health	3	1.3
Other communicable diseases	77	33.5
Others∗	39	17.0
Types of intervention		
Immunization	60	26.1
Pharmaceutical	54	23.5
Screening	35	15.2
Public health intervention excluding screening and immunization	16	7.0
Surgical	14	6.1
Others†	51	22.2

HIV indicates human immunodeficiency virus; NCD, noncommunicable disease; TB, tuberculosis.

∗ Others included physical disabilities, agronomic biofortification, and nondisease policy areas such as health financing mechanisms.

† Others included voucher systems, medical devices, taxation, expansion of access to a given service, aquaculture, and health financing mechanisms.

Most studies were single-country studies (80%). Most studies were conducted in the Sub-Saharan African region (39.1%), followed by South Asia (15.2%). Communicable diseases (eg, tuberculosis, malaria, human immunodeficiency virus) were the focus of most studies (59.1%).

Foundations, such as the Bill and Melinda Gates Foundation, were cited as the funding source most frequently (34.8%), followed by governments (26.1%). A considerable number of studies (20.0%) did not mention/report the source of funding, and 7.0% of the studies reported not having received any funding.

### Thresholds in Use

We found several CETs in use that can broadly be classified as (1) GDP based, (2) willingness to pay (WTP) or “demand side” thresholds, and (3) opportunity cost or “supply side” thresholds (see Box 1 for definitions).

Box 1 **Thresholds in use**

In this review, the 0.5× GDP per capita was classified as an opportunity cost CET. All authors that used 0.5× GDP per capita referred to opportunity cost threshold estimates and were thus categorized as opportunity cost thresholds in this paper. A total of 3 studies use opportunity cost thresholds. For instance, Olney et al^3^ estimate the CEA of different strategies used to improve HIV care outcomes along the cascade of care in Kenya and use 0.5× GDP as the threshold. Opportunity cost-based thresholds are often based on an estimate of the marginal productivity of the healthcare system that describe the amount of money in the present health system, needed to produce a unit of health due to the resource constraint. Two cross-country estimates from Woods et al^12^ (2016) and Ochalek et al^26^ (2018) have found that estimates average 0.5 GDP per capita, which is why this threshold is still defined against GDP per capita.

GDP-based thresholds refer to the 1 to 3× GDP per capita rule that is commonly referred to as WHO recommended threshold. If the ICER of an intervention is equal to or less than 1× GDP per capita per DALY averted, then the intervention is deemed highly cost effective and if the ICER is between 1 and 3× GDP per capita per DALY averted then the intervention is considered cost effective. Above 3× GDP per capita, the intervention is considered not cost effective.

Another method often referenced is the WTP CET, which is based on aspirational values, representing a specific view of what value ought to be placed on a unit improvement in health. It can be estimated using preference data collected through a WTP survey from the population to elicit the average maximum amount of money individuals are willing to pay for a unit of health gain.

CET indicates cost-effectiveness threshold; DALY, disability-adjusted life-year; GDP, gross domestic product; ICER, incremental cost-effectiveness ratio; WHO, World Health Organization; WTP, willingness to pay.

### Thresholds Used by Intervention and Study

GDP-based CETs are the most commonly (84.3% of studies) used CET decision rules in CEA studies in LMICs in the period studied. As noted in Box 1, this category includes only thresholds within the range of 1 to 3× GDP per capita. Notably, 3 studies use opportunity cost thresholds; 7.3% of studies did not state a rule. Others (0.9% studies) include articles that used regional thresholds (Price et al^19^ [2016]) and comparison of regional ICERs (Kaucley and Levy^20^ [2015]). Interestingly, we have found no study using WTP thresholds.

Additionally, we have found that some studies (eg, Nayagam et al^21^ [2016]) used multiple thresholds (GDP-based threshold and $240 per DALY).^21^

It is worth noting that not all studies need a CET. In this review, 14 studies found the interventions under consideration cost saving, dominated, or dominant. Please see Appendix 1 in Supplemental Materials found at https://doi.org/10.1016/j.jval.2021.08.014 for the table illustrating CET used by intervention and study.

### GDP-Based Thresholds

We further explored what GDP-based threshold was used (see Appendix 1 in Supplemental Materials found at https://doi.org/10.1016/j.jval.2021.08.014).

Moreover, 65.4% of studies used 1× GDP per capita, and 3× GDP per capita was used by more than a third of studies (34.2%).

### Recommendations on Cost-Effectiveness

Here, we cover the recommendation formulated by authors of the respective studies about cost-effectiveness. We show 2 main values: by intervention and by study (we look at whether a given study finds least 1 cost-effective intervention when several interventions are considered), 79.3% of interventions were found cost-effective. This rate was higher when considering studies rather than intervention: 85.2% of studies reported that at least one of the interventions under consideration was cost-effective.

Recommendations about cost-effectiveness were not stated in 11.6% of studies. For instance, Tolla et al^22^ (2016) highlighted the controversy surrounding the use of 1 to 3× GDP per capita, and on that basis, the authors decided not to issue a recommendation for cost-effectiveness. Only considering studies where a CET was applied and a recommendation was made (ie, excluding “not stated”), the proportion of interventions that were found to be cost-effective was 89.7% (see Appendix 2 in Supplemental Materials found at https://doi.org/10.1016/j.jval.2021.08.014).

### Recommendations on Cost-Effectiveness, by Type of Threshold Used

The recommendations on cost-effectiveness, by type of threshold used, are presented in Table 2.

**Table 2:** Recommendation on cost-effectiveness, by type of threshold.

By intervention	Recommendation	GDP-based threshold (n = 522)		Non-GDP-based threshold (n = 115)	
		Frequency	Percentage	Frequency	Percentage
	CE	472	90.4	33	28.7
	Not CE	50	9.6	8	6.9
	Not stated	0	0	74	64.4
	Total	522	100	115	100

By studies	Recommendation	GDP-based threshold (n = 194)		Non-GDP-based threshold (n = 36)	
		Frequency	Percentage	Frequency	Percentage
	At least 1 CE intervention	183	94.3	13	36.1
	No CE intervention	11	5.7	23	63.9
	Total	194	100	36	100

*Note.* Intervention section: only includes interventions whose CE recommendation was clearly stated. Interventions that were reported as dominant, dominated, or cost saving were excluded from the table above. Non-GDP-based threshold included opportunity cost CET and also included interventions where the CET was not stated.

CE indicates cost-effective; CET, cost-effectiveness threshold; GDP, gross domestic product.

We find that only 36.1% of studies using a non-GDP-based threshold reported at least 1 cost-effective intervention, versus 94.3% of studies using a GDP-based threshold. The proportion of studies not stating a recommendation was high in the non-GDP-based threshold category: 64.4% of studies.

## Discussion

The use of a CET is aligned with the goal of maximizing health in a budget constrained setting.^23^ By using thresholds that seem to be set too high and reject a small proportion of interventions, the probability of allocating resources to interventions that are not good value for money increases, resulting in displacement of health and net health losses to the society.^23^

This review reveals that several CET approaches are in use in LMICs, including GDP-based thresholds and opportunity cost-based thresholds. GDP-based thresholds remain the most frequently used CET in CE studies in LIMCs (84.3%), and we even observe an upward trend compared with the Leech et al^2^ review (66% from 2000 to 2015).

This is a surprising result given the numerous criticisms of the 1 to 3× GDP per capita thresholds,^9^^,^^10^ including recent warnings from WHO staff.^11^^,^^14^ This study finds that those criticisms seem to have had limited impact in discouraging their use, at least in published research. We found no record of local thresholds in our review. To further compound matters, we have found that in some countries where a local threshold exists, the GDP-based thresholds superseded the local rule. For example, Suraratdecha et al^24^ (2018) used 3× GDP per capita as a threshold in Thailand, despite the fact that there is an established threshold in the country (equivalent to 0.3× GDP per capita and 1.5× GDP per capita depending on severity).

Another surprising result is that 34.2% of studies applying GDP-based thresholds use 3× GDP per capita. Studies have criticized the use of 1 GDP per capita threshold in LMICs as being “too high” and 1 to 3× GDP per capita for having a wide cost-effectiveness range.^9^^,^^12^ As an illustration, for Kenya, the difference in cutoff between those 2 rules would mean applying 1816.5 or 5449.5 US dollar (based on 2019 GDP per capita) per DALY averted as a CET.^25^ If we consider estimates produced by Woods et al^12^ and Ochalek et al^26^ (2018) on health opportunity costs (which seem to average to 0.5× GDP per capita for every DALY averted), then it would suggest including introducing interventions that avert a DALY at 3× GDP per capita is likely to displace up to 5 times more health than the intervention creates. In other words, if an intervention averts a single DALY at 3× GDP per capita and the true cost of averting a DALY in the country is equal to approximately 0.5× GDP per capita, then the introduction of that intervention would in principle lead to a net loss of health equivalent to 5 DALYs averted.

Overall, we find that a high proportion of interventions (79.3%) were found to be cost-effective, and 85.2% of studies had at least 1 cost-effective intervention. There is no objective metric for assessing whether the proportion of negative recommendations found in this study is “too low.” Nevertheless, given the significant budget constraints, for example, in some Sub-Saharan African countries (where 39.1% of studies were conducted), we would postulate that this proportion of negative recommendations is too low, and therefore, the application of the 1 to 3× GDP per capita (without consideration of other evidence) may not enable decision makers to discriminate effectively between cost-effective and not cost-effective interventions. It is worth noting that factors other than the CET may lead to low proportion of negative recommendations (eg, publication bias); nevertheless, we were not able to investigate those in this study.

The reasons for the continued use of 1 to 3× GDP per capita as a threshold cannot be confirmed in this review; nevertheless, it should be noted that if ease of use (eg, the fact that it is a ready to use, off the shelf solution) makes it attractive, then the opportunity cost threshold (or 0.5× GDP per capita) is also similarly easy to use. We recommend, at the very least, that researchers include a justification and appropriate caveating when using 1 to 3× GDP per capita. Moreover, whenever a local threshold exists, there is no reason why it should not be used by study authors. It essential for CEA publications to have a clear and transparent use of appropriate thresholds and recommendations to allow such evidence to easily be used by the decision makers. Further research should be conducted to determine how decision makers use the CEA recommendation.

### Policy Implications

Together, these findings reinforce the view that 1 to 3× GDP thresholds are not a good rule for making recommendations about cost-effectiveness. Researchers should prioritize using local thresholds when available or explore alternatives to this rule. Funders and development agencies should consider discouraging its use in the future.

### Study Limitations

This article focused on studies that had reported DALYs; therefore, CETs based on quality-adjusted life-years or other health-related outcome measures were not included in this study. In addition, only 1 database was considered in identifying articles for inclusion. Although the GH CEA database is known for its comprehensiveness, there may be studies that are missed out because they were not uploaded onto the GH CEA database. Manual data recording (eg, on the threshold use or the recommendations) may have been prone to random errors. We only considered the use of CETs in published, English-language, research articles.

In addition, we were not able to review the use of thresholds by decision makers when making coverage decisions, because those are not always documented and may be difficult to identify.

## Article and Author Information

**Author Contributions:***Concept and design:* Kazibwe, Gheorghe, Ruiz, Chalkidou, Chi

*Acquisition of data:* Kazibwe

*Analysis and interpretation of data:* Kazibwe, Gheorghe, Wilson, Chi

*Drafting of the manuscript:* Kazibwe, Wilson, Ruiz, Chi

*Critical revision of the paper for important intellectual content:* Kazibwe, Gheorghe, Wilson, Ruiz, Chalkidou, Chi

*Statistical analysis:* Kazibwe, Chi

*Obtainingfunding:* Chalkidou

*Administrative, technical, or logisticsupport:* Kazibwe, Chi

*Supervision:* Ruiz, Chi

**Conflict of Interest Disclosures:** Dr Wilson is employed by the Bill & Melinda Gates Foundation, which includes funding work in health economic evaluations. Dr Chalkidou is an editor for *Value in Health* and had no role in the peer review process of this article. No other disclosures were reported.

**Funding/Support:** This study was funded by the 10.13039/100000865Bill & Melinda Gates Foundation through the International Decision Support Initiative Plus grant (grant number: INV-006987).

**Role of the Funder/Sponsor:** The funder had no role in study design, data collection and analysis, decision to publish, or preparation of the manuscript.

## Supplemental Material

Appendices 1 and 2
