# Supplementary material for: The Use of Cost-Effectiveness Thresholds for Evaluating Health Interventions in Low- and Middle-Income Countries From 2015 to 2020: A Review
Source: Value Health. 2022 Mar;25(3):385–9. doi: 10.1016/j.jval.2021.08.014 (PMC8885424; doi:10.1016/j.jval.2021.08.014)
Supplement: Appendices 1 and 2 [file mmc1.docx]

**SUPPLEMENTARY**

***Supplementary 1:*** Thresholds used by intervention and study

Table showing thresholds used by intervention and study

|  | **Threshold** | **Interventions** | **Studies** | |
| --- | --- | --- | --- | --- |
|  |  | **Frequency** | **Frequency** | **Percent** |
| **Type of Threshold** | GDP based Threshold | 522 | 194 | 84.3 |
|  | Opportunity cost | 22 | 3 | 1.3 |
|  | WTP | 0 | 0 | 0.0 |
|  | No Rule | 90 | 17 | 7.3 |
|  | Not applicable* | 76 | 14 | 6.1 |
|  | Other | 3 | 2 | 0.9 |
|  | **Total** | **713** | **230** | **100** |
| **GDP based Threshold*** | 1 x GDP per capita |  | 127 | 65.8 |
|  | 3 x GDP per capita |  | 66 | 34.2 |
|  | **Total** |  | **193** | **100** |

Note: Opportunity cost *CET includes 0.5x GDP per capita since the respective authors referenced published opportunity cost CET estimates.*

Not applicable* *includes the following: cost saving (4.4%), dominant (1.3%) and dominated (0.4%).*

*The table section for GDP based Threshold shows the proportion of the different GDP based thresholds used in studies*

*GDP based Threshold*: one study was not included in the table because it used regional GDP per capita. All studies included in the table above used country specific GDP per capita.*

Others (0.9% studies) includes articles that used regional thresholds and comparison of regional ICERs. Price et al (2016)^1^ referred to a “regional threshold of $6462 per DALY” to determine the cost effectiveness of pre-exposure HIV prophylaxis during pregnancy and breastfeeding in several countries in Sub-Saharan Africa. The regional threshold used was based on the average GDP per capita of the countries included in that study. Kaucley and Levy (2015)^2^ in their CEA of routine immunization and supplementary immunization activity for measles in Benin did not use any specific CET but compared their ICER with the ICER of similar studies that had been carried out in the region. A recommendation was made referencing other recommendations made by similar studies. Interestingly, we have found no study using WTP thresholds.

***Supplementary 2:*** Recommendations by authors

Supplementary 2 shows the recommendation formulated by authors of the respective studies about cost-effectiveness.

Table showing proportion of interventions that were reported as cost effective and studies that reported at least one intervention as cost effective

| **By interventions** | **Recommendation (N=637)** | **Frequency** | **Percentage** |
| --- | --- | --- | --- |
|  | Cost effective | 505 | 79.3 |
|  | Not cost effective | 58 | 9.1 |
|  | Not stated | 74 | 11.6 |
|  | **Total** | **637** | **100** |
| **By studies** | **Studies (N=230)** | **Frequency** | **Percentage** |
|  | Studies with at least one cost effective intervention | 196 | 85.2 |
|  | Studies without any cost-effective intervention | 34 | 14.8 |
|  | **Total** | **230** | **100** |

*Note: the sample size of interventions in this table is 637 as it excludes 76 interventions where a CET was not applicable (e.g. cost saving, dominant, and dominated).*

*Study section in this table includes all studies included in this review including those where the threshold was not applicable or not stated.*
